# Dark triad traits, study and power motives among medical students–A cross-sectional study at a German medical faculty

**DOI:** 10.1016/j.heliyon.2024.e32842

**Published:** 2024-06-11

**Authors:** Jasmin Bujok, Viktoria Witte-Humperdinck, Johannes Schulze, Daniela Ohlendorf

**Affiliations:** Institute of Occupational, Social and Environmental Medicine, Goethe University Frankfurt, Frankfurt am Main, Germany

**Keywords:** Dark triad, Multi-motive grid, Medical study, Traits of medical students, Implicit motives

## Abstract

**Background:**

A good physician should be empathic and altruistic, among other qualities. Therefore, the levels of socially undesirable personality traits (Dark Triad) as well as implicit motives of achievement, affiliation and power (Multi-Motive Grid) among medical students as future physicians were analyzed at two different points in their medical training.

**Methods:**

This study includes 380 medical students in their first year and 217 in their third year in Germany. All participants completed the Dirty Dozen (DD) and Multi-Motive Grid (MMG) questionnaires at the end of two different classes as paper-and-pencil tests. Relevant differences of the Dark Triad traits between the medical students and reference sample and the two different cohorts, as well as their implicit motives, the associations of Dark Triad traits and MMG components and gender differences of the Dark Triad traits were calculated.

**Results:**

There were no significant group differences between year one and year three medical students in narcissism, psychopathy and Machiavellianism (Dark Triad). There were no significant differences between the medical students and reference sample except in psychopathy. Male students scored significantly higher in the Dark Triad traits than female students. In the MMG, first-year students scored significantly higher levels in Fear of Rejection, and lower levels in Hope of Success and Hope of Power than the third-year students. Some associations were found between narcissism and Machiavelliansim with Hope of Success, Hope of Power and Fear of power.

**Conclusions:**

Dark Triad traits already appear to exist before the commencement of medical studies. These traits do not differ significantly between the medical students and reference sample; only a few MMG components seem to differ at different stages of their studies. This lack of differences between the medical students and validation cohort indicates that tests based on (undesirable) personality traits are not suitable criteria for the admission selection of medical students.

## 1 Introduction

Social skills such as altruism and empathy, as well as the communication skills of physicians, play an important role in the patient-physician interaction and, thus, are indispensable skills for future medical practitioners. For example, the patient-physician interaction has been rated negatively by patients when these skills were lacking in the physicians, particularly in terms of information provided about therapeutic alternatives or drug administration. [[1], [2], [3]]. Physicians demonstrating empathy and sensitivity toward patients were perceived as especially competent when it came to deliver (bad) news; their high empathy was rated positively by their patients. [4]. Therefore, the development of social skills and communicative abilities, as well as a special moral understanding, should represent a substantial part of a good physician, and should ideally also be part of the selection process for medical students.

Among German medical students, knowledge of these personality traits and their motives is limited. [5,6]. Concerning the motives to become a physician, Sönnichsen et al. [5] assessed the motivation of freshmen medical students at Marburg University by asking for the reasons, and perspectives, for their career choice. They found that the most important motives to study medicine were altruistic ones such as “wanting to work with and help other people.” Furthermore, in a study among medical students in Munich, intrinsic and extrinsic motivations were measured in the first, third and 6th year as well as in the “Praktisches Jahr” (the practical year, the last year in German medical school which consists of a one-year clinical internship). A change in motivation was shown for the high motivation “to help people” (intrinsic) at the beginning of their studies towards “taking up a secure profession”(extrinsic) and “acquisition of knowledge” (intrinsic) in their final year. [6]. Moreover, in another cross-sectional study among Lebanese medical students, the levels of academic motivation differed between the students in the five academic years. [7]. Other studies have also shown an association of higher motivation with early patient contact in medical school. [8,9]. However, the questionnaires used asked explicitly for motives to become a physician; no study addressed the expression of implicit motives and Dark Triad traits.

In terms of motive measurement it was shown that implicit motives are a different concept

to explicit ones. [10,11]. The self-report measures of motives, such as those used in most of the studies presented above, directly ask people about their motives and, thereby, assume that these motives are represented consciously and are, therefore, considered explicit motives. On the other hand, most unconscious aspects of our motivations are likely to be implicit and these can be measured by semi-projective tests such as the MMG. [12]. Contrary to self-reported motives, the use of semi-projective tests avoids socially desirable answers and, therefore, their use is important. [13]. While explicit motives are better predictors of conscious goal settings and attitudes, implicit motives are better predictors of long-term developments such as career choices, behavior types and other open life choices. [12]. Implicit motives are also associated with networking behaviors at work which are important for success in the chosen career domains. [14].

There are associations between empathy and the Big Five personality traits (extraversion, agreeableness, openness, conscientiousness and neuroticism) among medical students. [15,16]. For instance, Airagnes et al. [15] assessed the association between the Big Five traits and cognitive empathy in medical school. They found that cognitive empathy was positively associated with “extraversion” and “conscientiousness”, while negatively with “neuroticism.” Likewise, significant positive correlations between empathy and “openness to experience,” “conscientiousness,” “agreeableness” and “extraversion” were found in a multi-institutional study among Portuguese medical students. [17]. In a further cross-sectional study, weak and moderate correlations were found among Spanish medical students. [16].

Besides the Big Five, “negative” personality traits in medical students should also be taken into account. These include the traits of “narcissism,” “Machiavellianism” and “psychopathy,” subsumed under the term “Dark Triad”. [18]. This psychological model was developed by Paul and Williams in 2002 and represents three distinct but overlapping constructs which, as a common feature, share a low general agreeableness. [18,19]. All in all, the Dark Triad traits are so-called “maladaptive traits” that are characterized by a high degree of aggression and a lack of modesty and altruism. [20]. In the present study, narcissism is reflected as a “grandiose self-view,” Machiavellianism as “taking advantage of others” and psychopathy as “insensitivity to others.” [20] So far, knowledge about these traits among medical students is limited. Among Danish students of various study programs, economy students scored significantly higher in the “Short Dark Triad” (SD3) [21] than psychology students. The authors concluded that these personality traits may have influenced the choice of study programs if present at enrollment. [22]. Moreover, among medical students and physicians in Croatia, Machiavellianism declines progressively as their professional level increases. [23]. In a UK study, hospital employees reached significantly lower average values in the Dark Triad traits measured by the NPI [24], MACH-IV [25] and the LSRP [25] than non-medical professionals. [25]. However, when the subgroups were analyzed, the surgeons achieved higher values in narcissism and the nurses higher values in psychopathy. [25]. Consequently, it is reasonable to assume a connection between these characteristics in medical students and their later career preferences (specialist disciplines). Regarding gender differences of the Dark Triad traits, prior research has shown that men score significantly higher than women. [18,26,27]. Furthermore, in a study that examined the relationship between the Dark Triad and the Big Five with leadership Intention and the motivation to lead, it was shown that variance in leadership intentions was explained by narcissism over and above that explained by extraversion. [28]. Another study about the relationship between the Dark Triad traits, motivation at work measured explicitly, and burnout in the working context showed that not only do relationships exist between internal and external motivations but also that people with high levels of narcissism are resilient to burnout and are highly motivated. [29].

So far, associations between implicit motives measured by the MMG and psychopathology (e.g., panic disorders, agoraphobia and generalized anxiety disorder) have been studied. It has been shown that avoidance tendencies in implicit motives are associated with psychopathology. [11]. However, to the best of our knowledge, no literature exists about the associations between implicit motives and the Dark Triad traits within non-clinical populations, within the work context or among medical students.

Therefore, this study examines implicit motives as well as the socially undesirable Dark Triad personality traits among medical students in Germany. Since personality traits in general are seen as stable, long-lasting, and internally generated personality factors [30] (possibly existing before starting medical school), they should be similar between medical students in different

years, but may be changed by personality selection processes during the medical studies or

examinations. We hypothesizedthat the Dark Triad traits should not differ between freshmen and year three students (hypothesis one), which would classify Dark Triad traits as a

possible factor for medical student selection. We also assumed that there would be a lower Dark Triad trait level in medical students compared to the reference sample since hospital employees have shown significantly lower levels of Dark Triad traits than non-medical professionals (hypothesis two). With respect to the future career preferences of medical students, we further hypothesized that those students with a future career preference of surgery would score higher in narcissism (hypothesis three) and those with a psychiatry preference would score lower in all three of the Dark Triad traits (hypothesis four). Moreover, we aimed to replicate the findings of prior research regarding gender differences of the Dark Triad traits, with men scoring significantly higher than women (hypothesis five). In terms of motivation, since differences in motivation have been demonstrated to occur during the different stages of medical school training, we tested for differences in the MMG components during the medical studies, possibly exerted by competition in courses and examinations. Thus, our participants were surveyed during year one and year three in medical school. Students who were in year three in Germany would have recently passed their first high stakes federal examination, “Physikum,” which is considered as the most challenging examination and is then followed by the clinical studies. This phase includes much more patient contact and clinical experience which are both associated with increased motivation. Thus, we assumed that, having passed the examination and entered the clinical study phase, the fearful components of the MMG should be reduced and the hopeful components increased (hypothesis six).

Finally, to fill the literature gap about the relationship between the Dark Triad personality traits and implicit motives, we aimed to explore the associations between the Dark Triad traits and the MMG components.

## 2 Methods

Data for first-year students were collected following the mandatory course of “Medical Psychology and Sociology” in the first year of their pre-clinical medical study at the Goethe-University Frankfurt/Main, while third-year students were asked to self-report at the end of a mandatory course in pathophysiology. At the end of both courses, the students were informed about the study, its intentions and its voluntary nature of participation. Data collection was anonymous and could be denied or terminated by the students at any time. After consent, the survey was conducted as a paper-and-pencil test while the students were still present in class at the end of the course. Sociodemographic questions such as gender, age, graduation marks from high school as well as future job preferences (internal medicine, surgery, psychiatry and others) were included. The ethics committee of the Goethe-University Frankfurt considered no formal ethic approval necessary (protocol dated April 1, 2015). All methods were performed in accordance with the relevant guidelines and regulations (e.g., Declaration of Helsinki). Informed consent was obtained from all participants.

### 2.1 Participants

The sample consisted of 380 medical students from the first year and 217 medical students from the third year. All relevant demographic parameters and future specialty preferences are shown in Table 1.

**Table 1:** Demographic characteristics of the study cohorts.

Cohort	1st year	3rd year	Total
Total *N*	*N* = 380	*N =* 217	*N = 597*
	♀: 238, ♂:140	♀: 119, ♂: 98	♀: 357, ♂: 238
	No answer: 2		
Age range (years), mean	♀: 17–34, *M* = 20.46	19-38, *M* = 23.53	17-38, *M* = 21.76
	♂: 18–35, *M* = 21.26		
High School grades (“Abitur”) range, mean	1–3.4	1–3.5	1–3.5
	*M* = 1.48	*M* = 1.62	*M* = 1.53
Future specialty preference			
Surgery			N = 150
Internal medicine			N = 111
Psychiatry			N = 23
Others/I don't know yet			N = 280

### 2.2 Survey instruments

Both Dirty Dozen (DD) and Multi-Motive Grid (MMG) questionnaires were obtained from the publisher as part of a university license.

### 2.3 Dark triad

The Dark Triad was measured using the “Dirty Dozen” test by Küfner et al. [20] from 2015; this is the German version of the Dirty Dozen questionnaire by Jonason and Webster from 2010. [26]. Although the three characteristics of the Dark Triad traits, narcissism, psychopathy and Machiavellianism, can be assessed in more depth by individual survey instruments, the DD questionnaire provides a quick and easy method for capturing the main content of the three characteristics in their subclinical form. [20]. The questionnaire consists of twelve items with a nine-point Likert scale. Machiavellianism is measured by items 1, 4, 7 and 10, psychopathy by items 2, 5, 8 and 11 and narcissism by items 3, 6 9 and 12. [20].

As a reference, the data of validation study 1 by Küfner et al. [20] from 501 participants were used, the majority of which were students of all scientific faculties (354 female, 147 male; age 23.02 years, SD = 3.81).

#### 2.3.1 Multi-Motive Grid

**Fig. 1:**
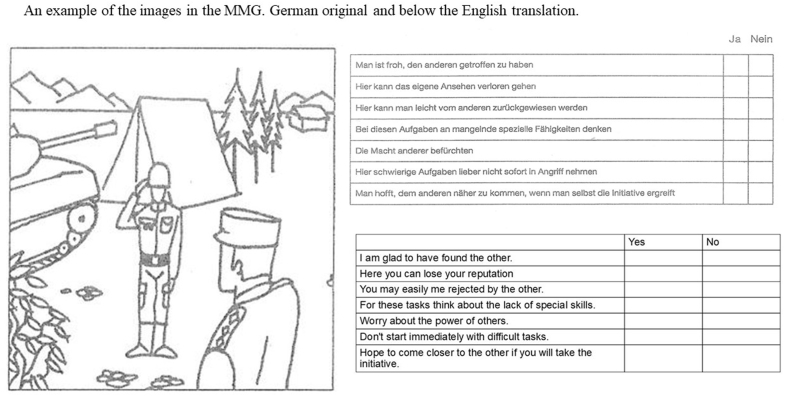
An example of the images from the MMG. The original images in German, are shown, with the English translations below.

The Multi-Motive Grid (MMG) from Schmalt, Sokolowski and Langens (revised in 2000) was used to measure the motives “Affiliation,” “Performance” and “Power” in their hopeful (positive) and fearful components (negative). [13]. This is a grid technique [12] that spontaneously and implicitly captures the above-mentioned motives with associations towards 14 ambiguous situations as shown in drawings; each picture includes statements to be affirmed or denied (one example is shown in Fig. 1). By means of this approach, answering according to social desirability or representation of explicit motives is minimized. [13,31]. Six motive characteristics are determined: Hope of Affiliation (HA), Fear of Rejection (FR), Hope of Success (HS), Fear of Failure (FF), Hope of Power (HP) and Fear of Power (FP). [12]. Participants were instructed beforehand that they would be shown some equivocal situations which represent everyday life situations, and that they should imagine to be one of the persons depicted. Among the situations, there are four to ten similarly structured statements, each of which should be answered spontaneously with “yes” or “no.” Unknown to the participants, each statement was either included in the evaluation or formed a filler item. For the motive values, the positively answered statements were summed using the test instructions. Validity and reliability were examined within the questionnaire construction for the German version.

The reference values for the MMG were taken from the test manual by Schmalt et al. [13] using results from the reference sample (*N* = 1919), from which the values are listed for all motives, both for the whole cohort and separately for both genders.

### 2.4 Statistical analysis

The present study is a cross-sectional study. Data were analyzed quantitatively using the statistical software SPSS (IBM, Version 27) and Excel (Version 1808).

To analyze group differences of the dependent variables in the two cohorts (freshmen and year three students) and future specialty preferences, multivariate analyses of variance (MANOVA) was conducted after prior examination of the outliers, distribution and homogeneity for the Dark Triad traits and the MMG components as dependent variables. Post-hoc univariate analysis of variance (ANOVA) was conducted and T-tests were used to examine gender differences in the Dark Triad traits. The significance level was set at 5 %. For the characteristics of narcissism, Machiavellianism and psychopathy, mean values were calculated from the corresponding items of the DD questionnaire. [20]. For the MMG components, values were calculated according to Schmalt et al. [13].

To compare the DD results of the medical students with the reference samples, the effect size was calculated as Cohen's d with Excel. The significance level was set at 5 %. Group differences of 0.5 scale points were considered relevant. A Cohen's d of 0.2–0.49 was considered a small effect, a value between 0.5 and 0.79 was considered a medium effect, and a d ≥ 0.8 was considered a large effect. Bonferroni correction was also applied.

Pearson correlations were calculated to assess the associations between the Dark Triad traits and MMG components.

## 3 Results

Inferential statistical analysis using MANOVA considering the Pillai trace revealed a significant effect of the year of affiliation on the combined dependent variables, F(9,574) = 2.079, p = 00.030, partial η^2^ = 0.032, pillai's trace = 0.032.

### 3.1 The Dark Triad traits among medical students

After considering the univariate analysis of variance, neither in narcissism (F(1,582) = 1. 91, p = 0.167), psychopathy (F(1,582) = 0.697, p = 0.404), nor in Machiavellianism (F(1,582) = 0.061, p = 0.804) significant differences were found between the two cohorts (Table 2). Thus, freshmen medical students neither differed significantly from the year three medical students in narcissism, nor in psychopathy, nor in Machiavellianism as assumed in our first hypothesis.

**Table 2:** Comparison of the Dirty Dozen (DD) traits between Frankfurt freshman students and third-year students with the reference sample (Küfner et al., 2015),^18^ and the comparison of gender differences among the total cohort. *p < 0.05, **p < 0.01, ***p < 0.001. *Legend: DD-N = narcissism, DD-M = Machiavellianism, DD-P = psychopathy.*

		*Mean value* ± *standard deviation*		
		Total	♀	♂
DD-N	Total (1st + 5th semester)	4.67 ± 1.62	4.42 ± 1.58***	5.04 ± 1.61***
	^1st^ semester (n = 380)	*4.60* ± *1.61*	*4.31* ± *1.59*	*5.08* ± *1.54*
	5th semester (n = 219)	*4.79* ± *1.63*	*4.64* ± *1.54*	*4.98* ± *1.71*
	reference sample (n = 501)	4.46 ± 1.71	4.43 ± 1.75	4.76 ± 1.57
DD-M	Total (1st + 5th semester)	3.48 ± 1.78	3.14 ± 1.61***	3.97 ± 1.89***
	^1st^ semester (n = 380)	*3.46* ± *1.83*	*3.06* ± *1.65*	*4.11* ± *1.91*
	5th semester (n = 219)	*3.51* ± *1.69*	*3.31* ± *1.53*	*3.76* ± *1.85*
	reference sample	3.21 ± 1.65	2.93 ± 1.54	3.87 ± 1.71
DD-P	Total (1st + 5th semester)	3.34 ± 1.57	3.06 ± 1.53***	3.77 ± 1.54***
	^1st^ semester (n = 380)	*3.31* ± *1.57*	3.07 ± 1.61	3.69 ± 1.43
	5th semester (n = 219)	*3.41* ± *1.58*	*3.03* ± *1.37*	*3.88* ± *1.69*
	reference sample	3.02 ± 1.47	2.73 ± 1.28	3.71 ± 1.64

When comparing the Dark Triad traits of medical students from those of the reference sample (hypothesis two), there were no relevant differences for narcissism (Cohen's d = −0.13) or Machiavellianism (Cohen's d = −0.157); only in psychopathy was a small effect size observed (Cohen's d = −0.21).

No significant difference in the dependent variable of narcissism with regard to the later career preference of surgery (hypothesis three) was found in the inferential statistical analysis (F(1,581) = 0.35, p = 00.56). Students who chose surgery as their later career preference did not score significantly higher in narcissism than those who had indicated another specialty as their future preference.

Similarly, the inferential statistical analysis using MANOVA showed no significant differences in the Dark Triad traits between those students who chose psychiatry and those who chose a different specialty as their future preference (F(3,579) = 0.549, p = 00.65) (hypothesis four).

There were statistically significant gender differences in all Dark Triad traits (hypothesis five). Female students scored lower in narcissism with a mean value difference of 0.62, (95%-CI [0.35,0.88]) t(583) = 4.59, p < 00.001, lower in psychopathy with mean value differences of 0.71, (95%-CI [0.45,0.96]), t(584) = 5.67, p < 00.001 and lower in Machiavellianism with a mean value difference of 0.83 (95%-CI [0.54,1.11]), t(584) = 5.67, p < 00.001 than their male fellows.

### 3.2 The Multi–Motive grid among medical students

Significant differences in the Multi-Motive Grid components of the 1st- and 3rd-year medical students (hypothesis six) were found in Fear of Rejection (F(1,582) = 5.037, p = 0.025), Hope of Success (F(1,582) = 9.059, p = 0.003) and Hope of Power (F(1,582) = 5.24, p = 0.022). First-year students scored significantly higher on Fear of Rejection (M = 5.64; SD = 2.69) than their third -year peers (M = 5.13; SD = 2.63). Moreover, first-year students scored significantly lower in Hope of Success (M = 6.72; SD = 2.41) than third -year students (M = 7.34; SD = 2.34). Similarly, first-year students achieved significantly lower levels in Hope of Power (M = 7.64; SD = 2.74) than third-year students (M = 8.16; SD = 2.53) (Table 3).

**Table 3:** The MMG's motive characteristics of the medical students and differences between the subgroups and reference sample. Significant differences between the first- and third-year cohorts are marked as * p < 0.05, **p < 0.01, ***p < 0.001. The standard deviation of the reference sample is not applicable.

		Affiliation		Performance		Power	
		Hope of affiliation	Fear of rejection	Hope of success	Fear of failure	Hope of power	Fear of power
raw values of the motive characteristic values	Total (1st + 5th semester)	6.21 ± 2.09	5.45 ± 2.68	6.95 ± 2.40	3.90 ± 2.35	7.83 ± 2.67	5.98 ± 2.69
	^1st^ semester	6.11 ± 2.14	5.64 ± 2.69*	6.72 ± 2.41**	3.96 ± 2.34	7.64 ± 2.74*	6.08 ± 2.74
	5th semester	6.37 ± 2.01	5.13 ± 2.63*	7.34 ± 2.34**	3.80 ± 2.38	8.16 ± 2.53*	5.70 ± 2.62
	reference sample	5.09 (n/a)	4.52	6.4	4.29	6.9	5.7

### 3.3 Relationship between the dark triad traits and the Multi-Motive Grid among medical students

There were low but significant correlations between Machiavellianism and Hope of Success (*r* = 0.12, p = 0.004), Fear of Power (*r* = 0.09, p = 0.023), and Hope of Power (*r* = 0.12, p = 0.004), and between narcissism and Hope of Success (*r* = 0.17, p < 0.001), Fear of Power (*r* = 0.14, p = 0.001) and Hope of Power (*r* = 0.20, p < 0.001). No significant correlations were found between psychopathy and the Multi-Motive Grid components (Table 4).

**Table 4:** Pearson's correlations (*r*) between the Dark Triad traits and the MMG's motive characteristics of the medical students. Significant correlations are marked as * p < 0.05, **p < 0.01, ***p < 0.001.

	Affiliation motive		Performance motive		Power motive	
	Hope of affiliation	Fear of rejection	Hope of success	Fear of failure	Hope of power	Fear of power
DD-N	0.039	0.014	0.171**	0.005	0.200**	0.136**
DD-M	0.019	0.040	0.120**	−0.030	0.120**	0.094*
DD-P	−0.021	−0.018	−0.001	−0.025	0.003	−0.034

## 4 Discussion

To the best of our knowledge, there are no publications about Dark Triad traits and implicit motives among medical students or about associations between those traits in general. Previous studies [17,32,33] have focused on the Big Five personality characteristics, which do not cover the Dark Triad traits, and do not distinguish between different motives like “fear” and “hope” motive. In this study, we found no relevant differences for the Dark Triad traits between first- and third -year medical students, hence, this confirms our first hypothesis. Traits usually are considered stable and, thus, Dark Triad traits should have been present before admission to medical school and so would not influence the student's choice of medicine. Consequently, we expected no difference in the Dark Triad traits between year one and year three students, which was confirmed by the results. The presence of stable and pre-existing traits is also supported by Vedel et al. [22] who found differences between students from different study subjects in the Dark Triad traits. These authors also concluded that the choice of study programs might be associated with these pre-existing traits, similar to the Big Five personality traits. In contrast, Bratek et al. [23] found a continuous decrease in Machiavellianism in medical students and physicians during their professional careers. Dark triad trait distribution apparently is not changed in our cohort by medical courses or examinations, although older studies suggested a low stability and major trait changes during college years. [[34], [35], [36]].

Comparing medical students to the test reference sample consisting of students from all faculties, again, no significant differences were found in narcissism and Machiavellianism. Only for psychopathy was there a small difference, with slightly higher values among medical students. Thus, our second hypothesis has to be rejected. It remains to be seen whether this difference in psychopathy is reproducible and/or relevant. Our findings are in contrast to the results of a study from the United Kingdom where all participating hospital employees, in contrast to non-hospital employees, had significantly lower expressions in all three components of the Dark Triad. [25]. Due to the lack of a control group in this study and the use of a different questionnaire, a direct comparison with our results is difficult; however, personality differences between hospital staff including nurses and (medical) students and physicians may explain these differences.

In terms of career choices, Jonason et al. [37] examined the influence of the Dark Triad traits on occupational preferences and found different trait patterns in relation to their preferred professional fields, i.e. higher scores in psychopathy correlated with practical occupations, narcissism with artistic, business or social careers, and Machiavellianism was associated with teaching or medicine but avoided caring or support. Bucknall et al. [25] confirmed high narcissism in surgeons compared to other specialties. No higher levels in narcissism, however, was confirmed in future surgeons compared to other specialties. In a Danish study of students from various disciplines [22], the economics students scored significantly higher in the Dark Triad traits than the psychology students; therefore, we assumed that among the medical students in our study, the ones who chose psychiatry as their later career preference would also reach significantly lower levels in the Dark Triad traits. In contrast to this expectation, students preferring psychiatry did not score lower than the rest in the Dark Triad traits, thus leading to the rejection of our fourth hypothesis. However, prior findings in terms of gender differences in the Dark Triad traits with men scoring higher than women [18,26,27] were replicated in our study and, thus, hypothesis five can be confirmed.

In terms of motive measurement, we found significant differences between the first- and third-year students. Against our expectations that all of the hopeful components would be increased and the fearful ones decreased in the later years, we only found significant differences for Fear of Rejection, with the first-year students scoring higher, and Hope of Success and Hope of Power, where the first-year students scored lower. Therefore, since our expectations were only partly met, hypothesis six had to be rejected. In the German medical studies, the first federal examination (“Physikum”) takes place after the 2nd year and is considered extremely difficult and a high stakes examination. Thus, students in their 3rd year participated in our study shortly after having successfully passed the “Physikum,” which possibly explains the increase in hope motives as well as the decrease in Fear of Rejection. Alternatively, the clinical study phase beginning in the third year included intense patient contact in multiple internships with a perceived higher relevance to their aspired career explaining the higher values of hope motives. The formation of informal student groups, often reinforced by learning groups in the preparation for examinations, may additionally also have contributed to the decrease in Fear of Rejection.

The motivation to become a physician has been previously investigated in German medical students albeit by different questionnaires concentrating on explicit motivation. [5,6]. Sönnichsen et al. [5] showed that German students at the beginning of their medical school career had high levels of study motivation; they had not yet been disillusioned by the theoretical topics like physics or biochemistry, nor by the realities of clinical medicine. These findings of a high motivation at the beginning of medical school could not be replicated in the present study measuring implicit motives. Furthermore, among first-year students, the scores in Fear of Rejection were significantly higher than among third-year students, while no other significant differences emerged in the remaining fearful components of the Multi-Motive Grid. One explanation could be the different survey instruments. While Sönnichsen et al. [5] measured motivation with questions like, “Why do I study medicine?” with different answer options on a five-point Likert scale, in the present study, implicit motives were measured by means of a semi-projective method. Thus, the tendency of social desirability is excluded by the semi-projective method but not by a direct questioning. Moreover, our study compared two different cohorts, whereas Sönnichsen et al. [5] only surveyed first-year students. Another reason for the lower levels in Hope of Success and Hope of Power among freshmen students in our study may be that they had not yet had many opportunities to gather personal experiences of study success at this point. Furthermore, in another study among medical students [6], comparing first-, second-, fourth- and sixth-year (practical year) students, it was shown that there was increased intrinsic motivation at the beginning of medical school that significantly shifted to extrinsic motivation towards the end of medical school. Since only explicit motivation was measured in these two studies and only implicit motivation in the present study, this may also explain the differences in the results and underline the concept of the separate measures of motivation between implicit and explicit methods.

Lastly, our findings demonstrate different associations between the Dark Triad traits and the MMG components. Low but significant correlations were found between Machiavellianism and Hope of Success, Fear of Power and Hope of Power. Narcissism was also significantly associated with Hope of Success, Fear of Power and Hope of Power. Otherwise, no significant correlations were found between psychopathy and the MMG components.

The present study also has some limitations. Along with the results of a study among hospital employees in England in which lower levels in the Dark Triad were found compared to non-hospital employees [25], we hypothesized that lower values in the Dark Triad traits should be expected in comparison to the reference sample by Küfner et al. [20] A control group with non-medical students would have been desirable as a possible methodological improvement; selection confounders may have influenced the mean values among different groups, thus hiding the true differences between medical and non-medical students. For this purpose, a randomized control group of both students (of all faculties) and non-student individuals from the overall population would be necessary. It is still unclear whether the Dark Triad traits and the Multi-Motive grid components change over time. However, the present study is a cross-sectional study and, therefore, is unable to measure individual changes. To compare specific traits at different times, and/or to estimate intraindividual changes, a longitudinal study would be necessary which also could estimate the malleability of these traits. Thus, future research should include individual changes in the Dark Triad traits and the implicit motives at different time points in a longitudinal study design. For better comparability of the traits among the different disciplines, future research should also incorporate additional control groups such as students of other disciplines, non-student cohorts or a balanced mix.

We also assumed a connection between the Dark Triad traits in medical students and their later career preferences (specialist discipline). However, specialist career choices occur far later than year one or three, and the students would have undergone multiple clerkships throughout their training, while medical socialization would also influencee their choices.

In summary, the present findings indicate a mostly similar degree of motivation and Dark Triad traits between medical students and other disciplines. Some specific results should be integrated into the evaluation of social skills during medical studies to encourage the students to reflect on their personal goals and motivations. The present findings extend our knowledge about dark Triad personality traits among medical students as they are comparable to the validation study reference sample. For implicit motives, the medical students showed higher values for Hope of Success and Hope of Power in year three than in year one, whereas Fear of Rejection was higher in year one than in year three. Thus, strengthening the medical students’ hopeful components in the earlier stages of medical school could be implicated for future teaching, although it should probably not be used in assessment tools to evaluate possible study programs.

Although narcissistic individuals can develop strong ambitions and accomplish great feats due to their elevated self-esteem [38], the inability to accept criticism [39] must be considered unfavorable for the medical profession since this may be accompanied by a lack of error introspection, emotional coldness and dominant aggressive behavior. [18]. Thus, it seems unlikely that a lack of altruism, emotional coldness [20] and a tendency for violent behavior and impulsivity [40] that are prototypical for subclinical psychopathy or Machiavellianism [41], would be advantageous for patient satisfaction.

This study was initiated by the international call for physicians with good soft skills, i.e. Communication, empathy, caring and altruism. However, in nearly all countries access to studying medicine is limited, often by academic performances in tests. It is unclear whether the soft skills currently desired cover different motives like hopeful and fearful implicit motives, or the Dark Triad traits. This situation of study admission by tests is common in many countries, the apparent discrepancy between tests and expectation of other qualities in the successful applicant seems to be most pronounced in medicine.

Our findings demonstrate that medical students are not significantly different from other students in their narcissism, psychopathy and Machiavellianism traits, while some implicit motives do differ. Thus, in order to support medical students to become good physicians hopeful motives should be strengthened and empathy training implemented at the early stages of medical school. These topics add to the Big Five personality traits, the study confirms that German medical students do not principally differ from the general student population.

## 5 Conclusion

First- and third-year medical students have comparable scores in the Dark Triad traits (narcissism, psychopathy and Machiavellianism) while some MMG components differed among the two cohorts of medical studies. No difference was found in comparison to the reference sample except for psychopathy. There were no significant differences in the Dark Triad traits among students who preferred surgery or psychiatry as their future career. Prior findings of gender differences in the Dark Triad traits were replicated. Passing the first federal examination in medical school may be a factor for the reduced Fear of Rejection and increased Hope of Power and Hope of Success among third-year students compared to freshmen; some of these expectations are supported by significant associations between the Dark Triad traits and implicit motives.

Our findings demonstrate that medical students are not significantly different from other students in their narcissism, psychopathy and Machiavellianism, while some implicit motives do differ at different stages of their medical studies. Thus, in order to support medical students, hopeful motives should be strengthened and empathy training implemented at the early stages of medical school.

## Funding details

There is no funding.

## Disclosure statement

The authors report that there are no competing interests to declare.

## Data availability statement

The datasets used and/or analyzed during the current study is available from the corresponding author on reasonable request.

## Ethics approval

The ethics committee of the Goethe-University Frankfurt considered no formal ethic approval necessary (protocol dated April 1, 2015).

## Permission to reproduce material from other sources

Not applicable.

## CRediT authorship contribution statement

**Jasmin Bujok:** Writing – original draft, Methodology, Investigation, Conceptualization. **Viktoria Witte-Humperdinck:** Writing – review & editing, Methodology, Investigation. **Johannes Schulze:** Writing – review & editing, Methodology. **Daniela Ohlendorf:** Writing – review & editing, Supervision, Methodology.

## Declaration of competing interest

The authors declare that they have no known competing financial interests or personal relationships that could have appeared to influence the work reported in this paper.
